# Mechanical characterization and optical microscopy of homemade slime and the effect of some common household products

**DOI:** 10.1038/s41598-022-07949-z

**Published:** 2022-03-10

**Authors:** Juveiriah M. Ashraf, Leia Nayfeh, Ammar Nayfeh

**Affiliations:** 1 Khalifa University, Abu Dubai, 127788 UAE; 2 Dunecrest American School, Dubai, UAE

**Keywords:** Gels and hydrogels, Polymer characterization, Mechanical properties

## Abstract

In this work, we demonstrate the synthesis of homemade slime and investigate how adding different household chemicals such as shaving cream and clay affects the chemical properties and hence the mechanical behavior. The purpose of this study is to instill scientific curiosity in young learners by establishing a relationship between a material’s chemical structure and its mechanical properties. Eight types of slime were studied: basic slime (borax with glue), slime with the addition of: (a) shaving cream, (b) clay, (c) shaving cream and clay together, (d) baking soda, (e) cornstarch, (f) hand soap, and (g) toothpaste. It was found that basic slime has a Young’s Modulus of 93 MPa while adding shaving cream and clay increased the modulus of elasticity to 194 and 224 MPa respectively. Adding thickening agents such as baking soda and corn starch increased the modulus to 118 and 110 MPa respectively while the incorporation of foaming agents, for example, hand soap and toothpaste rendered the sample very gelatinous. The Young’s modulus of samples C and D was the highest recorded and this is attributed to the presence of clay, which is relatively the stiffest material from the choice of additives used in this study. The results were supported by FT-IR spectroscopy which showcased the formation of different chemical structures of the slime with the added chemical agents.

## Introduction

Commercial slime is popular among young children for fun and educational purposes, and it can also be useful in showcasing school students how mixing different substances can change the chemical nature and thus the elastic properties of materials. It can thus be a vital technique in developing analytical thinking in children with regards to the chemical bonding of materials at the microscale and how it is directly interlinked with the mechanical properties of a material (macroscale). Such studies can form the basis of developing hands-on scientific demonstrations which aid in students’ analysis and observation of chemical processes: from building a hypothesis to designing simple experimental methods, carrying out practical instigations, and finally making inferences from the results obtained. Students can also be exposed to the presence of microscopy techniques like scanning electron microscopy (SEM) and chemical characterization techniques such as Fourier transform infrared spectroscopy (FT-IR).

Slime is a non-Newtonian fluid^1,2^, i.e., unlike Newtonian fluids whose viscosity remains unchanged when strain is applied, the viscosity of slime increases with applied temperature and pressure. More specifically, slime is a dilatant; under stress it undergoes shear thickening, and the material dilates/expands. Other examples in this class of materials are quicksand, printer’s ink, and starch solutions. When squeezed, slime exhibits flexibility since the cross-links between the polymers are able to break and form again quickly. However, if the same fluid is pulled apart suddenly, it will be ruptured. This unpredictable response of the material to applied strain makes it an interesting material to be studied for school-going children. Investigating its properties will allow students to discern the difference between Newtonian fluids such as water or honey and non-Newtonian fluids such as slime. They can also be compared to pseudoplastic fluids (also a type of non-Newtonian fluid) in which the viscosity increases with increasing strain applied. Examples of such fluids include paints, nail polish and tomato sauce.

The simplest slime is synthesized by mixing poly(vinyl alcohol) (PVA) with sodium tetraborate (borax), which is a salt of boric acid. The reaction between PVA and borax forms cross-links between polymer chains due to the creation of weak bonds to the OH groups of PVA^3,4^. The three-dimensional network polymers formed as a result lead to the viscoelastic nature of the fluid that gives slime its specific texture. The sodium tetraborate interlinks with the PVA through hydrogen bonds^5^ or reversible covalent bonds^6^ to form “di-diol” complexes, which constitute two diol units and one borate ion, yielding the gel-like material. The linkage is proposed to be of two types: (a) where the linkage between diols and borate ions is based on both, a physical and chemical nature^7^ and (b) where only a chemical bond exists in the form of cross-links between PVA polymers and borate ions^8–11^. Few studies have researched the chemical nature of cross-linking borax with PVA^10^, the effect of employing salts for coagulating slime^1^, and explained the phenomena of polymeric binding using slime^2^. To the best of our knowledge, however, no study has been done on determining the mechanical properties of homemade slime along with interlinking it to its chemical nature. In this work, we analyze homemade slime using optical imaging, Fourier transform infrared spectroscopy, and mechanical characterization techniques to evaluate the bonding and elastic properties of slime and investigate the effect of adding some common household materials.

## Synthesis of slime

**Figure 1:**
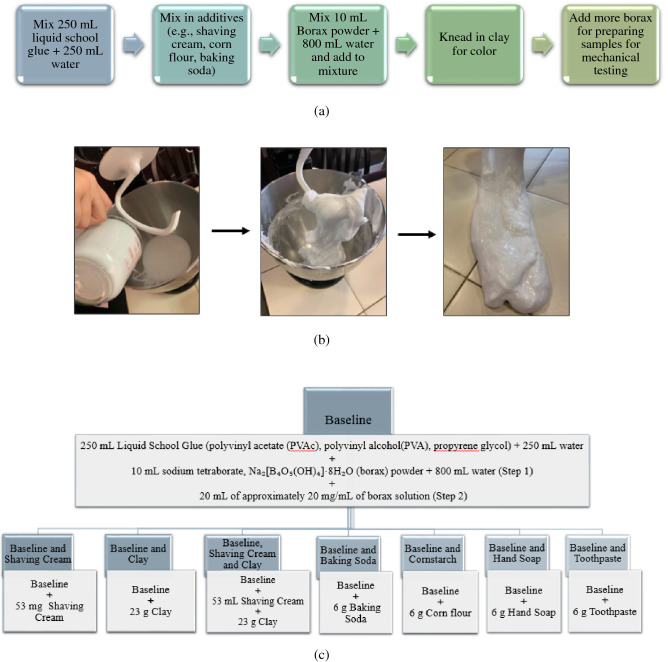
(**a**) Step-by-step process to make slime, (**b**) intermediate steps showing how adding borax leads to stiffening of slime, and (**c**) process flow to make eight different types of slime.

The slime was prepared in four ways: baseline (A), baseline with shaving cream (B), baseline with clay (C) and baseline with shaving cream and clay (D). The baseline was made by combining a mixture of 250 mL of liquid school glue (which is an emulsion of poly(vinyl acetate) (PVAc)), poly(vinyl alcohol) (PVA), and propylene glycol) and 250 mL water with a mixture of 4.2 g sodium tetraborate, Na_2_[B_4_O_5_ (OH)_4_]·8H_2_O (borax powder) with 800 mL water which was prepared in a separate bowl. The borax was added in part (8 increments) and mixed using a mixing tool, the slime becoming stiffer with each addition (step 1). Both mixtures were kneaded well until the slime was formed. To the baseline, 53 mg of shaving cream (SC), (mainly water, stearic acid, and lanolin) was added to make mixture B. Mixture C was prepared by adding 23 g of kid’s modelling clay (green) to the baseline while mixture D was made by mixing shaving cream and clay to the baseline in the same amounts as in mixture B and C. Samples E, F and G and H were prepared by adding 6 g of each: baking soda and corn flour, foaming handwash and toothpaste to the baseline slime respectively. Additional borax solution was added to make the samples ready for mechanical testing (step 2 for adding borax). 20 mg mL^−1^ concentration borax solution was prepared and 20 mL of this was added to all samples. For the mass measurements, an electronic kitchen scale is used with a precision of ± 1 g while for the volume measurements of 800 mL and 10 mL, laboratory beakers are employed that have a precision of ± 100 mL and ± 5 mL respectively. Figure 1a illustrates the step-by-step process to synthesize slime, b depicts the formation of slime by the addition of borax and c shows the process flow to make eight different types of slime. This a simple framework for making slime, with a lot of room for innovation; the recipe can be modified as needed with different household products as are desired to be studied.

## Characterization

**Figure 2:**
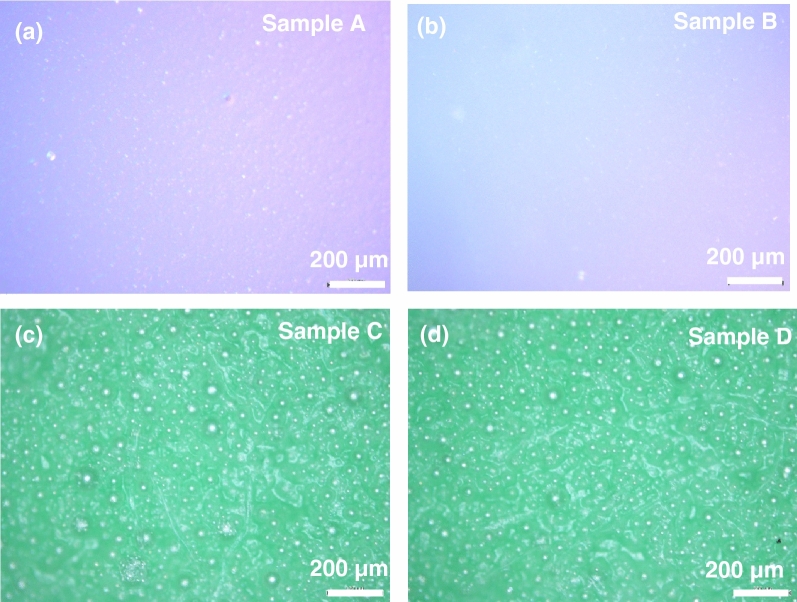
Optical microscopy images for: (**a**) baseline (Sample A), (**b**) baseline and shaving cream (Sample B), (**c**) baseline with clay (Sample C), and (**d**) baseline with shaving cream and clay (sample D).

**Figure 3:**
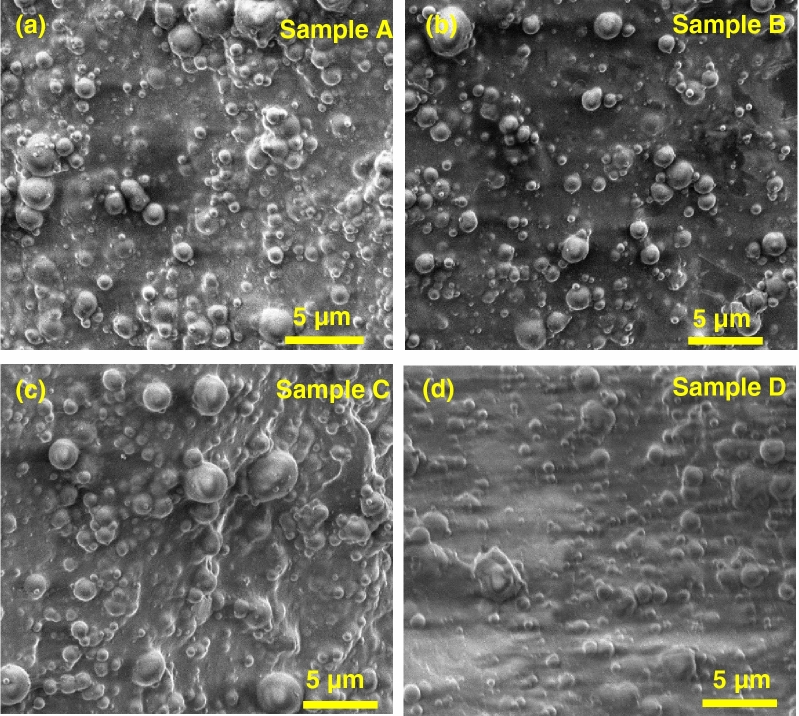
SEM images of samples A–D at a magnification of around × 10,000.

The slimes were tested by scanning electron microscopy and optical microscopy to observe the internal structure of the specimens and mechanical testing to evaluate the elastic modulus. For the optical microscope images, a clear distinction is seen between samples A and B vs. C and D. The latter two samples appear green due to the presence of clay in the specimens. The images showed samples C and D (with added clay) to have larger bubbles which may be due to high water-absorbing and moisture-retention capabilities of clay while samples A and B appear to have slightly smoother surface at the microscale (see Fig. 2). Scanning electron microscopy (SEM) images (taken via Quanta 250 ESEM) taken with a spot size of 2.5 and a voltage of 2.00 kV are shown in Fig. 3 which depict the slime samples at higher magnifications (approximately 10,000×). These can help students perceive how gel-like materials appear on the microscale compared to the conventional optical microscope, and students can observe the formation of bubbles for hygroscopic (water-absorbing) materials like slime. Scanning electron microscopy can also determine the density of bubbles in the sample. Sample A and B has smaller bubbles while sample C has coalesced bubbles. Moreover, we notice that specimen D has less bubbles. This could be related to the change of hygroscopic nature of the samples once the clay added. The slime images under the SEM look similar due to the operating conditions of the microscope, i.e., high vacuum under which formation of bubbles appear, which makes it difficult to discern between the bubbles in specimen versus the bubbles formed due to low pressure in the microscope. Nevertheless, optical microscopy and SEM images can be observed by elementary students to better understand the nature of slime at different microscopic levels and inculcate the importance of using different characterization techniques in materials science.

**Figure 4:**
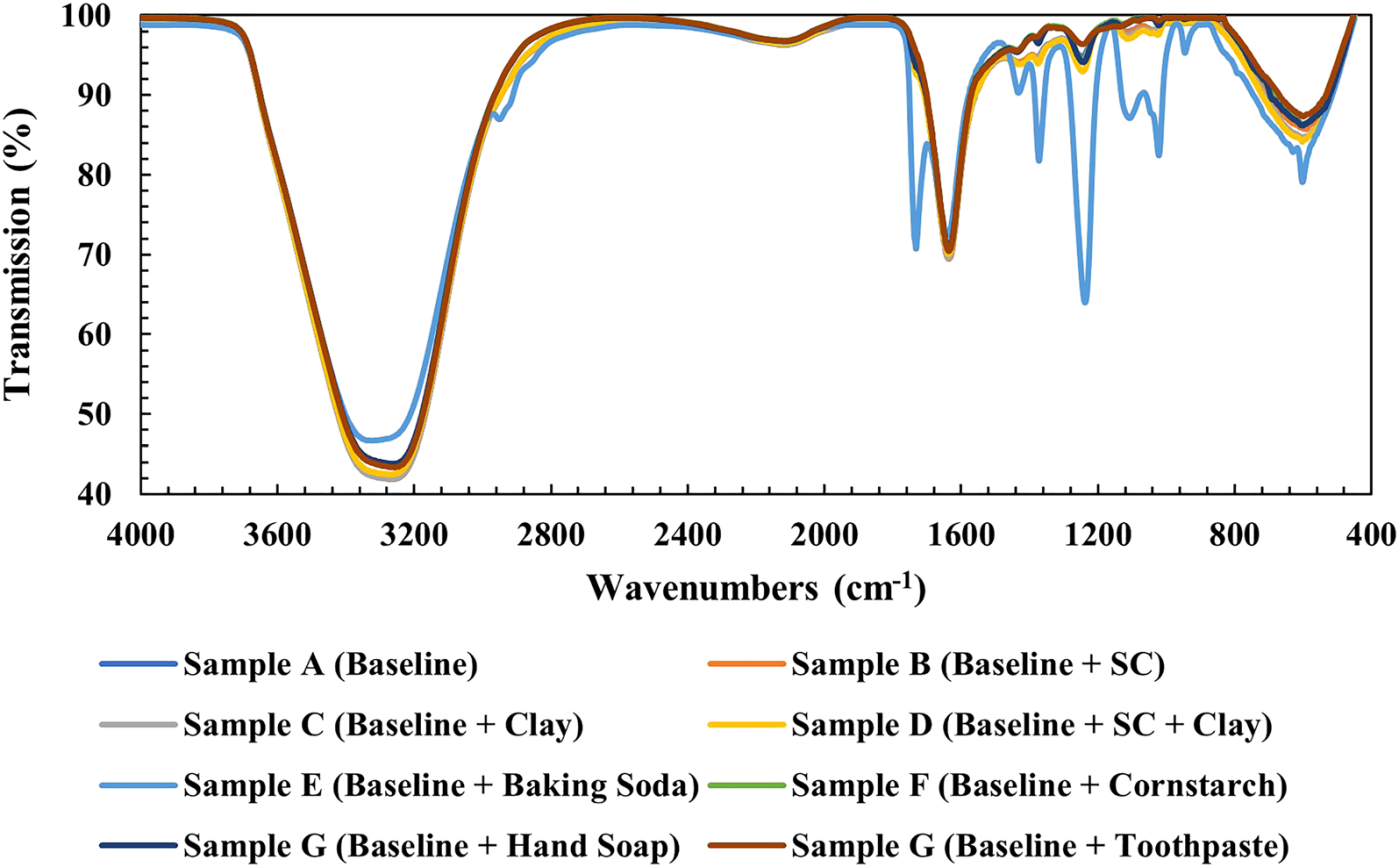
FT-IR spectra obtained for samples A–H.

To determine the presence of functional groups, FT-IR spectra for wavenumbers from 460 to 4000 cm^−1^ were taken for the samples using the Bruker Vertex 80v FT-IR spectrometer (Fig. 4). For the test, a small amount of the specimen is taken by spatula and placed over the IR-ATR source point then irradiated to get the FT-IR spectra. Samples require blending with potassium bromide (KBr) by 1:99 ratio. In simple terms, every material on earth absorbs light which is not detectable by naked eye. Here, FT-IR can be used to bring to the students’ attention the importance of using such instruments capable of detecting the absorbance/transmission of light of different materials with respect to their functional groups. The cross-linking between the PVA hydroxyl groups and borax induce the formation of ester groups which are validated by the bending of B–O–B linkages within borate networks, for which the representative FT-IR peak around 597 cm^−1^ is clearly seen in Fig. 4^12^. The sharp peaks at 3315 cm^−1^ were detected due to the presence of O–H stretching vibration—an indication of unreacted OH^13^, (but is slightly broadened here due to the H bonding interaction). It is difficult to discern the difference in IR absorption from C–H (hydrogen) bonding as it is not only present in PVA, but also in the starch of clay and stearic acid of shaving cream. The functional groups arising from the borate ions are present in all samples. The tetrahedral BO_4_ group produces a peak at approximately 1377 cm^−1^ while the asymmetric stretching relaxation around 1432 cm^−1^ of B–O–C (from the BO_3_ trigonal group)^13^. An interesting change in the FT-IR spectrum is observed after adding baking soda (sample E); different peaks appear are detected, specifically at 948, 1025 (independent peak of NaHCO_3_), 1117, 1240, 1373, and 1436 (analytical peak of Na_2_CO_3_) cm^−1^. Although it is be expected that the other additions, for example, toothpaste (which contains high amount of fluoride), hand soap or cornstarch to also show a discrete pattern in the spectrum, no significant variation is observed, and here we suggest the use of other material characterization techniques such as Raman and X-ray diffraction (XRD) to be used to get an elaborate description with regards to the chemical bonding and crystallinity of the samples respectively.

**Figure 5:**
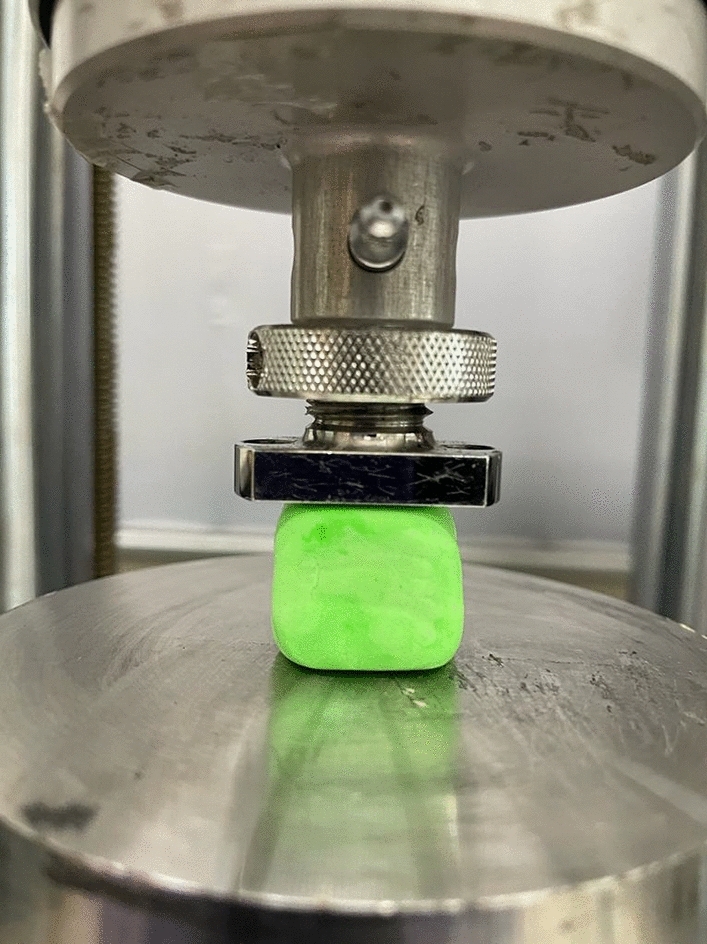
A slime specimen (sample C) ready for mechanical compressive tests.

Mechanical testing was carried out using the Instron 5940 Universal Testing System. The size of samples used in mechanical compression was approximately 2 cm × 1 cm × 1 cm. The load of the displacement control is performed at a rate of 0.3 mm min^−1^ (or 0.005 mm s^−1^). Samples were placed in the center to ensure uniform loading. The static loading of the samples was carried out using a 2 kN load cell and the stress–strain curves were produced in real-time by the Instron Bluehill 3 software. The mechanical compression test works by applying compressive pressure on a cuboid sample which results in stress–strain diagrams being produced for the specimen. The elastic modulus is calculated by dividing the stress (which is force over area) by the strain (the change in length over the original length). From the test, different material properties can be calculated such as elastic limit, proportional limit, yield point, yield strength, and, for some materials, compressive strength. Below is an image of a specimen ready for compression testing (Fig. 5).

**Figure 6:**
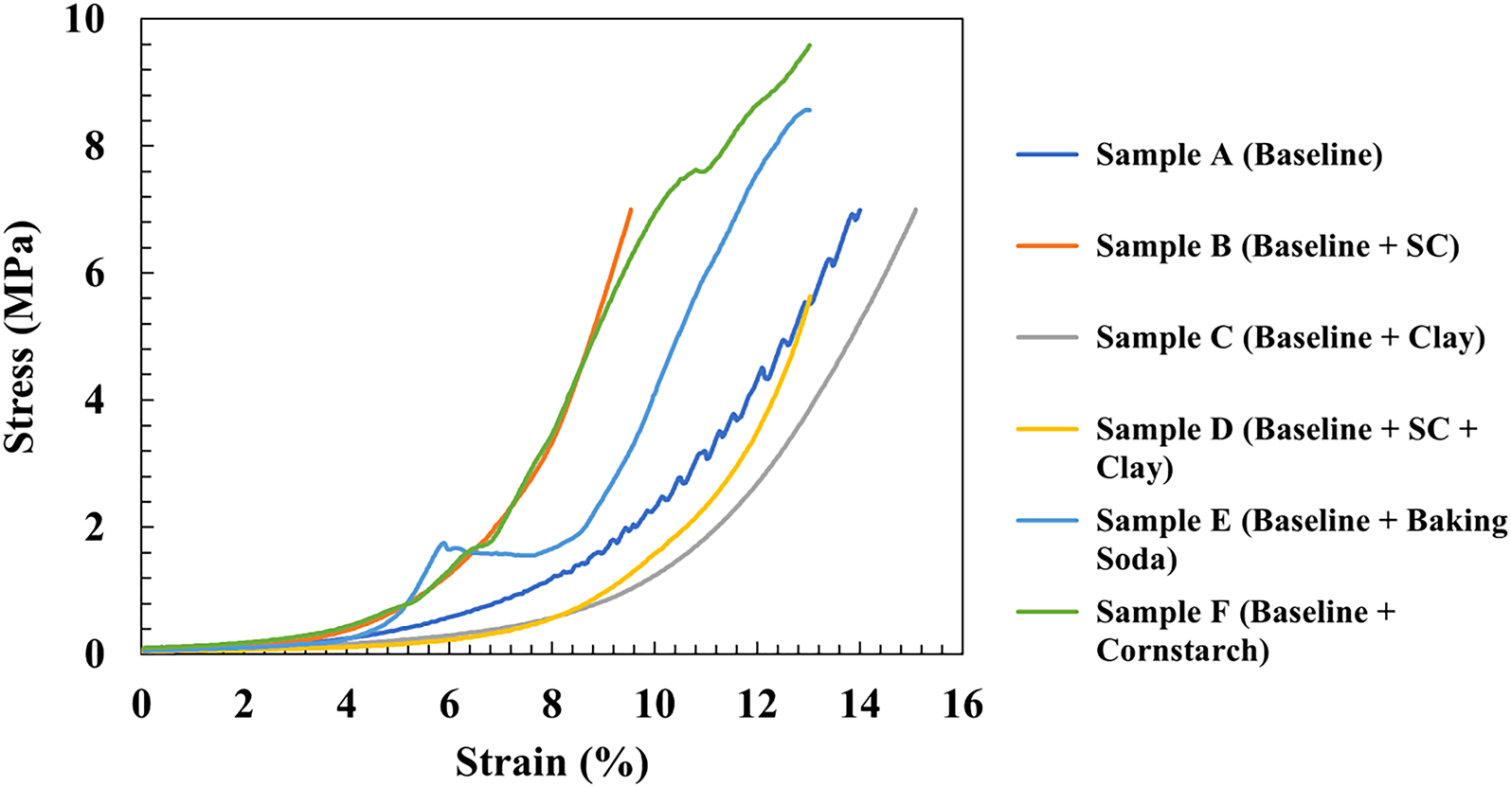
Stress–strain curves of samples A–F.

Mechanical testing results proved the high elasticity of the slime samples: specimen A had an elastic modulus of 93 MPa while adding shaving cream (specimen B) increased the modulus to 194 MPa. As is known, a higher elastic modulus signifies a stiffer material, i.e., it stretches less when pulled and vice versa, thus the addition of shaving cream decreases the elasticity of the slime samples. Similarly, incorporating clay with into the baseline (specimen B) also decreased the flexibility of the slime (increasing the Young Modulus by more than twice to 224 MPa), making it more manageable and keep its shape after deforming. Consequently, adding both clay and shaving cream also resulted in an overall stiffer material with an elastic modulus of 229 MPa. Moreover, samples E and F were also tested. It was found that adding baking soda (sample E) also increased the modulus to 118 MPa while adding corn starch (sample F) increased it to 110 MPa. It is evident from these 2 samples that the slime becomes stiffer on adding cornstarch and baking soda, which are in fact used in food as thickening agents. The stress–strain curves are represented below in Fig. 6.

Here it is crucial to mention that the samples G and H (with hand soap and toothpaste additives respectively) could not be tested for their mechanical properties due to their highly gelatinous nature. This could perhaps be attributed to the combination of foaming and surfactant agents added to soaps and toothpaste which lead to the slime’s inability to hold shape. This is an important observation in the slime study and could bring to the students’ attention the distinct nature of thickening agents such as corn starch and baking soda as well as cleaning agents (such as soap and toothpaste). This activity is vital to direct students to make connections with existing data to what they already know. Another inference that can be made is that the Young’s modulus of samples C and D still remain the highest due to the addition of clay which is relatively the stiffest material from the choice of additives used in this study. To summarize, the Young’s Modulus is a measure of the interatomic bond force, i.e., the stronger the atomic bonding is, the larger the Young’s Modulus^14,15^. When different chemicals are added, the bonds (as observed in some cases by the FT-IR spectra) between the material changes and hence causes a shift in the elastic modulus and consequently the stretchability of the samples.

## Additional information

**Figure 7:**
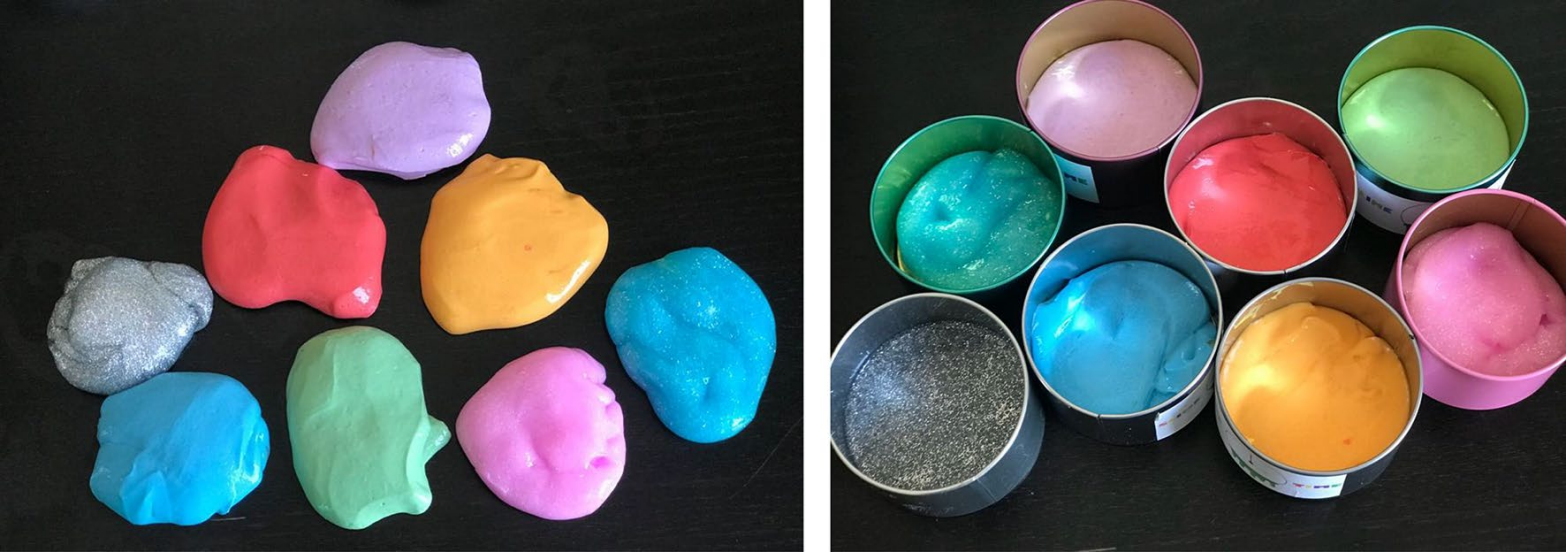
Adding colored clay to make different colored slime.

Different colored clay was added to enhance the visual appeal of slime as is seen in Fig. 7 below. Clay was used as opposed to traditional food coloring for a stronger consistency and to maintain the texture and mechanical properties of the slime as have been studied above.

## Slime kits

**Figure 8:**
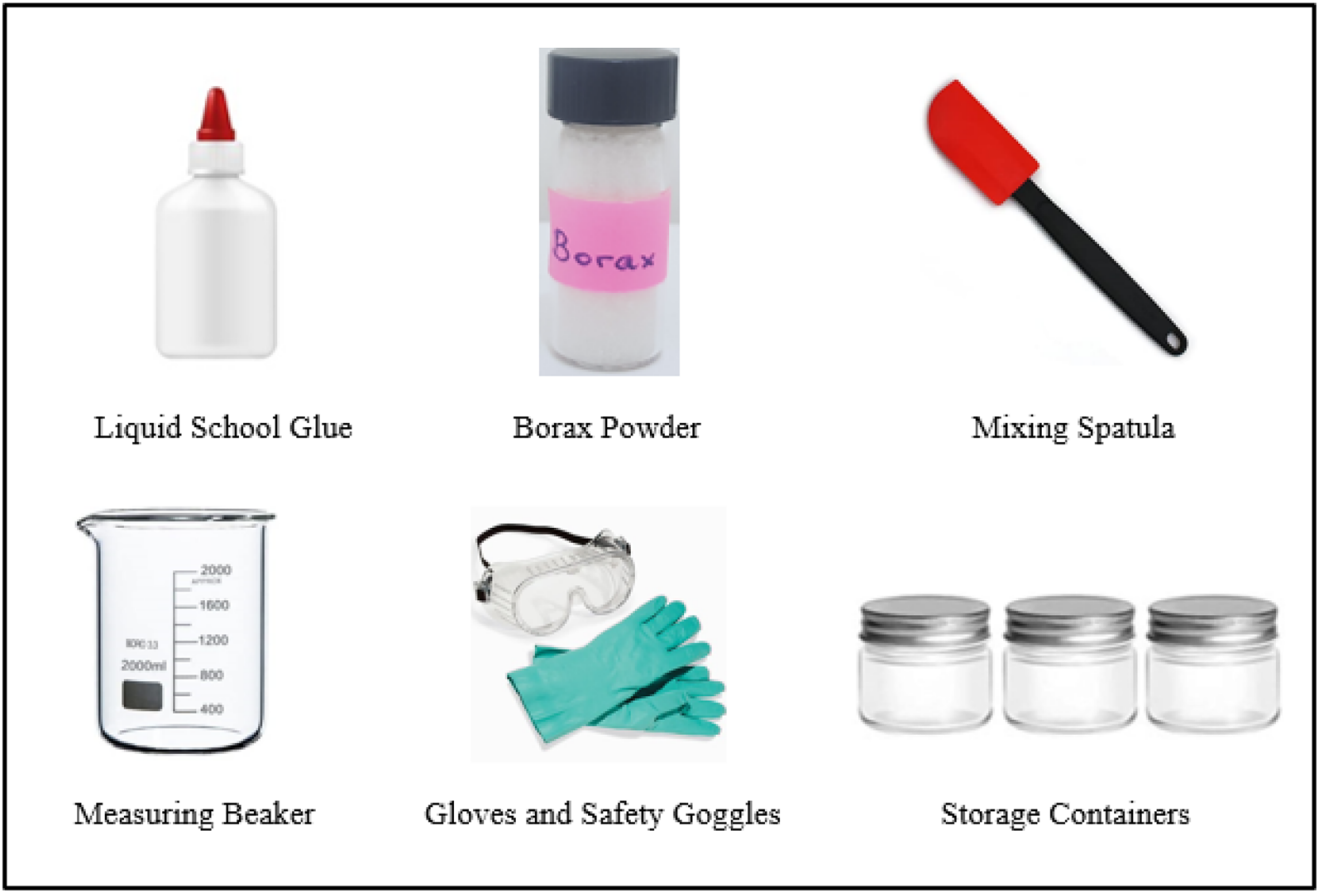
Slime kit for kids: ingredients and supplies.

In terms of education, we propose slime kits that can be prepared for younger students with the ingredients and supplies to synthesize slime and an easy-to-follow instruction manual which would facilitate the learning process. The slime kit would include: liquid school glue, borax, a beaker for measuring, a spatula for mixing, small containers to store the slime, gloves and safety goggles and an instruction manual. A few items of the slime kit are showcased below in Fig. 8. This would allow students to investigate by adding different products at home or school and observe the physical effect on slime. The use of borax can cause serious eye irritation while swallowing larger amounts may cause gastrointestinal symptoms such as abdominal discomfort, nausea, vomiting and diarrhoea. It is important thus to monitor children below the age of 12 while such experiments are being carried out and the wear of gloves is necessary during handling.

## Conclusion

To summarize, the mechanical properties of eight types of slime were studied by adding different chemicals to baseline slime and interlinked with their chemical characteristics for educational purposes. The samples were also studied under optical and scanning electron microscopy to help students visualize the material at the microscale. It was found that base slime has a good elasticity with a Young’s Modulus of 93 MPa while adding shaving cream and clay increased the stretchability with Young’s Modulus to 194 and 224 MPa respectively. Adding thickeners such as baking soda and corn starch increased the modulus to 118 and 110 MPa respectively while the integrating foaming agents (hand soap and toothpaste) resulted in gelatinous samples. We believe that studying this material will be interesting for students to identify different properties of materials on a fundamental level, induce the application of new skills and concepts, and encourage students of applying alternative scientific explanations.

## Data Availability

The datasets generated during and/or analyzed during the current study are available from the corresponding author on reasonable request.
